# Erosion protection conferred by whole human saliva, dialysed saliva, and artificial saliva

**DOI:** 10.1038/srep34760

**Published:** 2016-10-05

**Authors:** T. Baumann, J. Kozik, A. Lussi, T. S. Carvalho

**Affiliations:** 1 Department of Preventive, Restorative and Pediatric Dentistry, University of Bern, Freiburgstrasse 7, CH-3010, Bern, Switzerland

## Abstract

During dental erosion, tooth minerals are dissolved, leading to a softening of the surface and consequently to irreversible surface loss. Components from human saliva form a pellicle on the tooth surface, providing some protection against erosion. To assess the effect of different components and compositions of saliva on the protective potential of the pellicle against enamel erosion, we prepared four different kinds of saliva: human whole stimulated saliva (HS), artificial saliva containing only ions (AS), human saliva dialysed against artificial saliva, containing salivary proteins and ions (HS/AS), and human saliva dialysed against deionised water, containing only salivary proteins but no ions (HS/DW). Enamel specimens underwent four cycles of immersion in either HS, AS, HS/AS, HS/DW, or a humid chamber (Ctrl), followed by erosion with citric acid. During the cycling process, the surface hardness and the calcium released from the surface of the specimens were measured. The different kinds of saliva provided different levels of protection, HS/DW exhibiting significantly better protection than all the other groups (p < 0.0001). Different components of saliva, therefore, have different effects on the protective properties of the pellicle and the right proportions of these components in saliva are critical for the ability to form a protective pellicle.

A wide range of prevalence data show that dental erosion is a common condition in developed societies. For adults (aged from 18 to 88 years), prevalences ranging from 4 to 100% have been reported^1^. Dental erosion is defined as a chemical dissolution of dental hard tissues that, in contrast to caries, does not involve bacteria^2^. It starts at enamel, the outermost layer of teeth, which consists mainly of calcium-deficient carbonated hydroxyapatite. In contrast to pure hydroxyapatite, part of the phosphate (PO_4_^3−^) and hydroxide (OH^−^) is replaced by carbonate (CO_3_^2−^), and part of the calcium (Ca^2+^) is replaced by sodium (Na^+^) or magnesium (Mg^2+^) in the crystal structure of this mineral^3^. These substitutions in the crystal lattice weaken the enamel structure, making it more susceptible to acidic dissolution than pure hydroxyapatite. Most acids relevant to erosion are weak acids, which dissociate in water into hydrogen ions (H^+^) and their respective anions^3^, while some of the acid molecules remain undissociated^4^. While the H^+^ will directly dissolve the apatite mineral surface, the undissociated form of the acid will also significantly contribute to enamel dissolution by penetrating enamel pores faster than the dissociated form because of its lack of charge. Once within the enamel, the molecule dissociates and the newly formed H^+^ carries on the dissolution of mineral^5^,^6^. Enamel dissolution from dental erosion is, therefore, a dynamic process starting with a softening of the enamel surface followed by surface loss^7^. Once surface loss occurs, the mineral cannot be replaced, so erosion at this later stage is irreversible and can result in dentine exposure, and even pulp exposure has been reported in epidemiological studies in children^1^.

Human saliva is the most important natural factor that is able to prevent acidic demineralisation and support remineralisation of the dental surface in different ways^8^. On the one hand, it can directly dilute, neutralise and buffer the acids or hinder them from reaching the dental surface. On the other hand, it can remineralise enamel because of its inorganic components. After erosion, ionic interactions between the demineralised tooth surface and saliva can occur, mostly with calcium, phosphate and fluoride ions^8^. A multitude of artificial saliva formulations and remineralizing solutions containing only inorganic components are able to remineralize erosive lesions to a certain extent^9^,^10^,^11^, although a complete remineralization is unlikely^7^.

Another way that saliva protects against erosion is by the formation of a salivary pellicle on the tooth surface. It is formed by adsorption of peptides and proteins onto the tooth surface. Pellicle formation starts immediately after toothbrushing because of ionic and hydrophobic interactions, as well as van der Waals forces, between the proteins and the enamel surface. It is a selective process, as only a specific subset of salivary proteins is present in the pellicle^12^. Calcium- and phosphate-binding peptides and proteins from saliva, especially statherins and acidic proline-rich proteins, can bind with high affinity to the calcium and phosphate on the tooth surface^13^. Additionally, the calcium-binding domains of these proteins can maintain calcium ions near the enamel surface and act as a reservoir^12^. Therefore, the salivary pellicle can simultaneously regulate the uptake and release of calcium and phosphate between the tooth surface and saliva. In this way, the pellicle acts as a semipermeable membrane and maintains the integrity and mineral homeostasis of the enamel surface^8^,^12^.

Many studies have shown that the natural salivary pellicle can partially protect the enamel surface from changes owing to acidic attacks^12^,^14^. However, the preventive potential of the salivary pellicle is limited and varies with each individual; the composition of the pellicle playing an important role in this variability^15^,^16^. It has been reported that whole saliva with a high level of calcium and phosphate helps prevent erosive demineralisation compared to dialysed, ion-depleted saliva^12^,^17^. The importance of the right pellicle composition was demonstrated in a study analysing the pellicle composition of erosion patients, which showed that pellicles of such patients contain a lower level of calcium and proteins such as statherins^13^. However, other studies do not show any correlation between salivary ion content and susceptibility to erosive tooth wear, so there is still some controversy about the actual effect of salivary ions and proteins on erosive demineralisation^8^,^18^,^19^. The aim of the present *in vitro* study was to investigate the differences in erosion protection that is conferred by treating enamel with natural saliva, dialysed saliva containing only salivary proteins and artificial saliva containing only salivary ions, to gain further insight into the importance of the different components of saliva for the formation of a protective pellicle.

## Materials and Methods

### Preparation of the enamel specimens

A total of 75 enamel specimens were prepared from human molars. The teeth were selected from a pool of extracted teeth, which had been extracted by dental practitioners in Switzerland (no water fluoridation, 250 ppm F^−^ in table salt) and were stored in 2% chloramine T trihydrate solution. Patients were informed about the possible use of their teeth and consent was obtained. The experiment was carried out in accordance with the approved guidelines and regulations of the local ethics committee (Kantonale Ethikkommission: KEK), which categorized the teeth as “irreversibly anonymized” because they had been pooled. The roots of the selected teeth were removed using an Isomet^®^ low speed saw, and the crowns were cut into buccal and lingual halves. Using two planar parallel moulds, the halves were embedded in acrylic resin. The thinner moulds (200 μm thick) were removed and the teeth were ground and polished using a Knuth Rotor machine with abrasive silicon carbide paper discs of grain size 18.3 μm, 10 μm and 5–6 μm. After removing the thicker moulds, they were further polished for 1 min with a 3 μm grain diamond paste on a polishing cloth under constant cooling. The specimens were stored in a mineral solution (1.5 mmol/l CaCL_2_, 1.0 mmol/l KH_2_PO_4_, 50 mmol/l NaCl; pH 7)^20^ until being used in the experiment. Immediately prior to the start of the experiment, they were additionally polished with a 1 μm grain diamond paste for 1 min under constant cooling. Between the polishing steps and after the final polish, all slabs were ultrasonicated for 1 min in tap water and rinsed. The specimens were randomly distributed into 5 treatment groups (n = 15, Table 1): humid chamber (control, Ctrl group); human whole stimulated centrifuged saliva (HS group); artificial saliva (AS group); HS dialysed against artificial saliva (HS/AS group); HS dialysed against deionised water (HS/DW group).

### Collection of human whole stimulated saliva

Stimulated saliva was obtained from adults aged between 20 and 30 years with no active caries. They were instructed not to eat or drink anything except water for 2 h before saliva collection. Stimulated saliva was collected into chilled vials while chewing on paraffin wax for 10 min. Immediately after collection, the saliva was pooled and centrifuged for 20 min at 4 °C (4000 g). After centrifuging, the supernatant was divided into aliquots of 20 ml, which were stored at −80 °C. The donors provided their informed oral consent to use the saliva for research purposes. Since the saliva was pooled, it was categorized as “irreversibly anonymized”, and the experiment was carried out in accordance with the approved guidelines and regulations of the local ethics committee (Kantonale Ethikkommission: KEK).

### Dialysis of human whole stimulated saliva

Dialysis was performed with a Mega Pur-A-Lyzer^TM^ Dialysis Kit with a membrane cut-off of 1 kDa (PURG10020, Sigma-Aldrich). Human saliva was dialysed against water or artificial saliva. Aliquots of 20 ml of human saliva were thawed and dialysed. Dialysis against artificial saliva (AS; 1.5 mM Ca(NO_3_)_2_, 0.90 mM KH_2_PO_4_, 130 mM KCl and 60 mM Tris buffer; pH 7.4^21^) was carried out in 2 l of AS for a total of 48 h at 4 °C. AS was exchanged after 2, 8, 16 and 24 h. Dialysis against deionised water was carried out in 5 l of deionised water for a total of 72 h at 4 °C. Water was exchanged after 2, 8, 16, 24, and 48 h. After dialysis, new aliquots of 1.85 ml were prepared and stored at −80 °C until further experiments were performed.

Although it is known that freezing and thawing of saliva can cause certain proteins to precipitate^22^, we did neither observe any precipitates nor turbidity after thawing the saliva. In any case, as all saliva prepared for the different groups were frozen and thawed, precipitation would have affected all groups equally, so differences between the groups caused by freezing and thawing can be excluded.

Saliva collection and dialysis resulted in the four different kinds of saliva used in this study: human whole mouth stimulated centrifuged saliva (HS); artificial saliva (AS); HS dialysed against artificial saliva (HS/AS); HS dialysed against deionised water (HS/DW).

### Analysis of calcium and inorganic phosphate concentrations

Total calcium (Ca) concentration in all solutions was determined using an atomic absorption spectrometer. Lanthanum nitrate (0.5%, lanthanum nitrate hexahydrate: La(NO_3_)_3_·6H_2_O) was added to the solution to eliminate the interference of other ions^23^.

Total inorganic phosphate (P_i_) was analysed using the method of Chen *et al*.^24^. A total of 2 ml of diluted solution was mixed with 2 ml of a phosphate reagent (2% ascorbic acid, 0.5% ammonium heptamolybdate, 0.6 M H_2_SO_4_), stored for 90 min at 37 °C, allowed to cool to room temperature (24 °C), and analysed using a photometer.

### Analysis of total protein concentration

The total protein concentration was determined colourimetrically using a Pierce^TM^ BCA Protein Assay Kit (23227, Thermo Scientific) with bovine serum albumin as a standard. The assay was performed in a 96-well micro plate, using triplicates of each sample and standard. The plates were read at 570 nm using an ELx808 Absorbance Reader.

### Incubation – erosion cycles

The experiment consisted of four incubation – erosion cycles. One cycle consisted of individually immersing the specimens for 1 h in 1.8 ml of the respective incubation solution, in a shaking water bath (70 rpm) at 37 °C (Gesellschaft für Labortechnik mbH, Burgwedel, Germany). In the case of the Ctrl group, the specimens were left in a humid chamber for 1 h at 37 °C. Afterwards, the specimens were rinsed with deionised water for 20 s, dried with oil-free air for 5 s, and surface hardness (SH) was measured.

The specimens were then submitted to an erosive challenge, which consisted of individually immersing the specimens in 10 ml of citric acid (1%; pH 3.6) in a shaking water bath (70 rpm) for 1 min at 25 °C (P-D Industriegesellschaft mbH Prüfgerätewerk, Dresden, Germany). After the erosive challenge, the specimens were rinsed with 100 ml of tap water for 20 s and dried with oil-free air for 5 s. SH was measured again, while the citric acid solutions were labelled and stored for later calcium analyses.

The examiner performing the SH measurements and calcium analyses was blind to the solutions. In total, the specimens underwent 4 cycles of incubation and a total of 4 min of erosion.

### Surface hardness measurement

Surface hardness (SH) measurements were carried out initially and after each incubation, as well as after each erosive challenge. The initial SH value was labelled SH_0_. Thereafter, odd numbered values (SH_1_, SH_3_, SH_5_, SH_7_) represent values after incubation periods, while even numbered values (SH_2_, SH_4_, SH_6_, SH_8_) represent values after erosions. SH measurements were carried out on a Fischerscope HM2000 XYp using a Vickers diamond under a pressure of 50 mN for 15 s and results were expressed in Vickers Hardness Numbers (VHN). For each SH measurement, a total of seven indentations were made parallel to each other, spanning an overall distance of 200 μm. Subsequent measurements were performed 100 μm from the measurements of the previous step. The mean value from the seven indentations at each step was considered for analysis. The changes in enamel hardness between the initial measurement (SH_0_) and the following steps were calculated as percentage change (∆SH) and used for statistical data analysis and interpretation. ∆SH was calculated using the following formula:

∆SH = (SH_i_/SH_0_) × 100, where SH_0_ is the initial measurement, and SH_i_ is the hardness value after the i^th^ measurement (after the i^th^ incubation in a solution or after the i^th^ erosion).

### Analysis of calcium release into the citric acid

After each erosive challenge, the amount of calcium released into the citric acid was analysed. Calcium was analysed as described before, and the measured calcium concentrations were normalized to the corresponding enamel surface areas of the specimens. To calculate the surface area, we used a light microscope (Leica, M420) at 16x magnification connected to a camera (Leica, DFC495). Using the software program IM500, the contour of the exposed enamel window was traced and the surface area of each specimen was calculated.

### Statistical analysis

Using an identical protocol, a preliminary study showed that specimens kept in a humid chamber presented a 55.3 ± 2.9% (mean ± sd) decrease in SH, whereas specimens incubated in human saliva presented a 52.3 ± 1.6% decrease in SH after 4 experimental cycles (effect size of d = 1.28). Considering an effect size of d = 1.3, an α error (type I) of 0.05, and a power (1 − β) of 0.80, we calculated a sample size of 11 samples per group (actual power 0.81; t = 2.09). Therefore, we used a sample size of n = 15 specimens per group.

The normality of the data sets was checked using graphical methods as well as Shapiro Wilk’s test. Both outcomes SH and Ca were not normally distributed (p < 0.0001). The main effects of the whole-plot factor group and time and their interaction were thus tested using non-parametric time-related ANOVA^25^. The resulting p-values were corrected for multiple testing with Holm’s method.

Initial absolute values of SH were analysed by Kruskal-Wallis test, revealing no differences between the groups. It was therefore excluded as variable in the further analyses. For SH, the percentage change (∆SH) was then used as the variable in the statistical analysis.

In the case of significance in the global test, post-hoc analysis was performed with Kruskal-Wallis tests for simultaneously comparing all different groups and then with Wilcoxon-Mann-Whitney tests for pair-wise comparisons. P-values in this section were not corrected for multiple testing owing to the explorative nature of this part of the analysis. The level of significance was set to 0.05.

## Results

### Surface hardness

**Figure 1:**
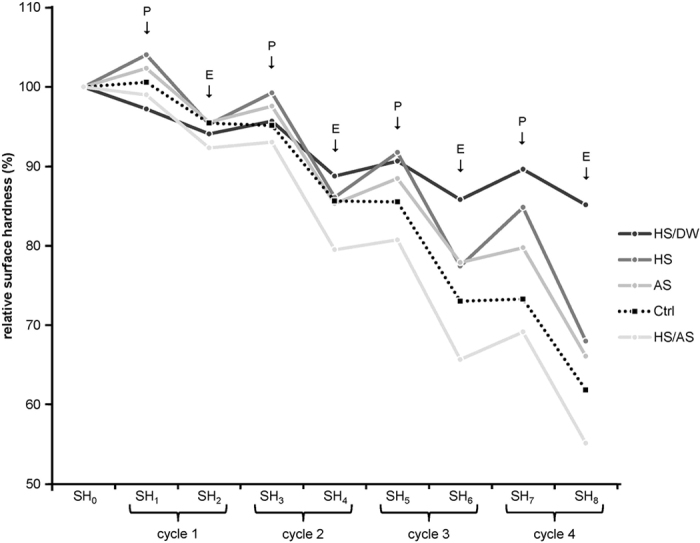
Change in mean relative SH for all groups during incubation - erosion cycles. **P** marks measurements after saliva incubation periods, **E** marks measurements after erosions.

SH was measured nine times: initially (at baseline – SH_0_) and after each subsequent incubation and erosion (SH_1-8_). The overall SH_0_ mean ± standard deviation was 504.2 ± 40.2 VHN, ranging between 444.8 and 612.7 VHN. Figure 1 shows the relative SH values for all five groups throughout the entire experiment. In general, SH values decreased in all groups after every erosive challenge. The incubation periods of 1 h generally led to an increase in SH values in all groups, except for the Ctrl group. This increase in SH was most evident in the HS group. However, the preceding hardness could not be restored, as the decrease in SH values after the erosive attacks was greater than the increase during the next incubation period. Both the degree to which SH values decreased (after the erosive attacks) as well as the degree to which SH values increased (after incubation) appeared to escalate with each experimental cycle.

The SH values of the HS group showed the greatest increase in SH after each incubation, increasing by 4%, 4%, 6%, and 8%, for SH_1_, SH_3_, SH_5_, and SH_7_, respectively, compared to the values before incubations. However, SH also decreased considerably in the HS group after each erosion step, with drops of 9%, 13%, 15%, and 17%, for SH_2_, SH_4_, SH_6_, and SH_8_, respectively, compared to the values before the erosion step. The latter decreases in SH values were comparable to those in the HS/AS group.

**Figure 2:**
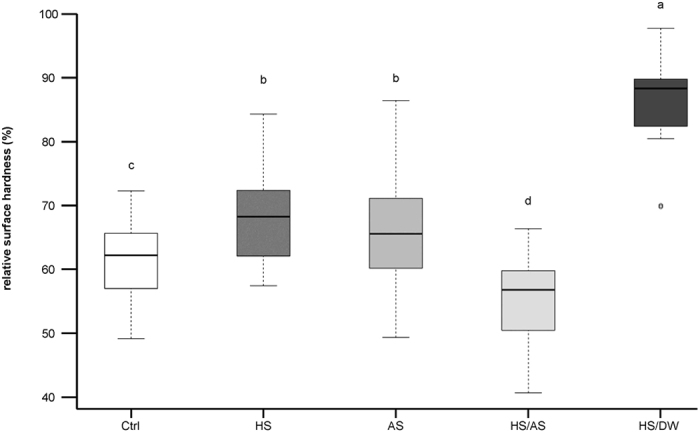
Final remaining relative SH after all experimental cycles for the different groups. SH decreased significantly less in the HS/DW group than in all the other groups. Different letters indicate significant differences between the groups (Wilcoxon-Mann-Whitney test, p < 0.05).

The HS/DW group exhibited the mildest decrease in SH values, leading to the highest SH values at the end of the experiment, and the HS/AS group exhibited the lowest SH values, significantly lower than the other groups from the SH_4_ measurement onwards. Figure 2 shows the relative SH values at the end of the experiment. The SH in the HS/DW group decreased from 100% to an average of 85.5 ± 7.6%, which was a significantly lower decrease than in the other groups (p < 0.001). The HS and AS groups had SH values decreasing from 100% to 68.0 ± 8.3% and 66.3 ± 9.8%, respectively, and they were not significantly different from each other (p = 0.513). They were, however, significantly different to the Ctrl group (62.1 ± 6.5%) and to the HS/AS group (p < 0.05). The latter presented the greatest decrease in SH, with values decreasing from 100% to 55.3 ± 7.0% (p < 0.05 compared to all other groups).

### Calcium release during erosion

**Figure 3:**
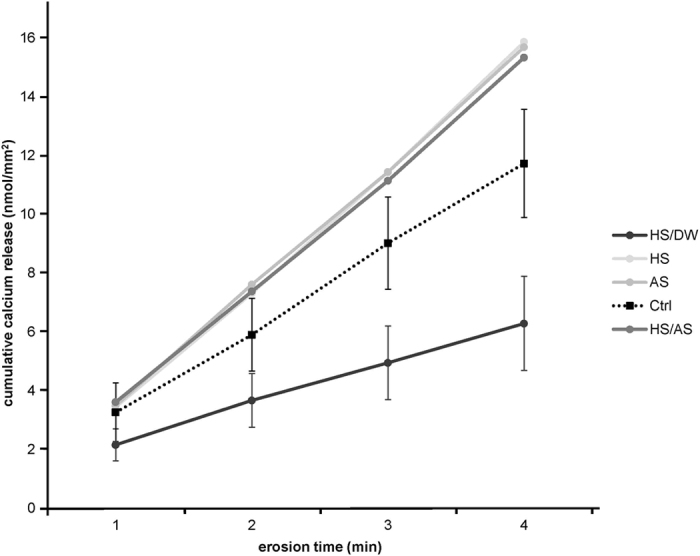
Mean cumulative calcium released (±SD) for all groups during erosion cycles. For reasons of clarity, the SD is only displayed for the HS/DW and the Ctrl group.

**Figure 4:**
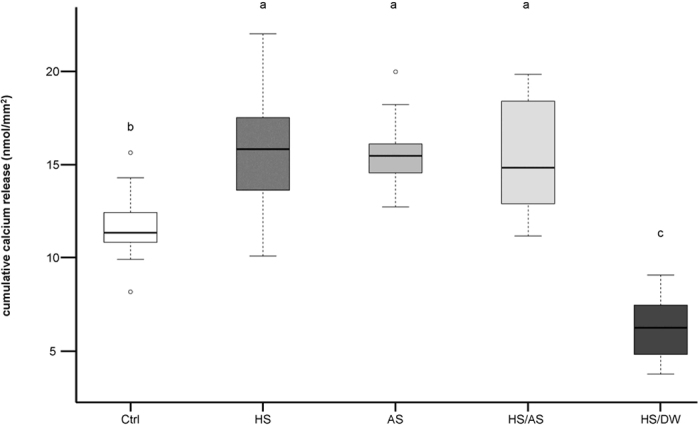
Total amount of calcium released after all experimental cycles for the different groups. The HS/DW group released significantly less calcium than the Ctrl group. Different letters indicate significant differences between the groups (Wilcoxon-Mann-Whitney test, p < 0.001).

The cumulative calcium release for all erosion cycles is presented in Fig. 3. From the first cycle on, the HS/DW group presented the lowest calcium release, while no differences were observed between the other groups. Figure 4 shows the total amount of calcium released after all cycles. Significantly different amounts of calcium were released in some of the groups. The HS/DW group clearly released the least amount of calcium (6.28 ± 1.60 nmol/mm^2^), followed by the Ctrl group (11.73 ± 1.84 nmol/mm^2^), and they both released significantly less calcium (p < 0.001) than the HS group (15.86 ± 3.14 nmol/mm^2^), the AS group (15.69 ± 1.80 nmol/mm^2^), and the HS/AS group (15.33 ± 2.98 nmol/mm^2^). However, no significant differences were observed in the pattern of calcium release for HS, AS and HS/AS groups throughout the entire experiment.

## Discussion

The saliva used in this experiment was collected from many donors and pooled to avoid any bias that could arise from using saliva from only one donor^26^. This pooled saliva was then used to form two additional groups: one group was dialysed against artificial saliva, resulting in an equal ion content between artificial saliva and human saliva, and another group was dialysed against deionised water, resulting in a solution with very low ion concentrations and mainly salivary proteins. Since salivary proteins and peptides bind calcium and phosphate ions, it was not possible to completely remove these ions from saliva using dialysis (Table 1). Nevertheless, the concentrations of these ions were greatly reduced, and we considered the resulting HS/DW as deprived of free ions.

For dialysis, we chose a membrane with a molecular weight cut off of 1 kDa. This would allow only the diffusion of ions and molecules smaller than 1 kDa, so the main protein content would remain in the dialysed solution. However, some salivary peptides are smaller than 1 kDa and may have diffused out of the saliva. Furthermore, some salivary proteins might stick to the dialysis membrane and be partly removed from the dialysed saliva. Since pellicle formation is a specific process, a possible minor partial removal of some proteins was tolerated as it should not affect the final pellicle composition. Hence, HS/AS and HS/DW had basically the same protein content since they were both dialysed, but different ion compositions (Table 1), and they both had lower protein content than the undialysed HS. Moreover, despite the small differences between the dialysed salivas and HS, comparisons can still be drawn.

In addition to the dialysed salivas and HS, we also used artificial saliva (AS) to assess only the effect of the present ions. The ionic concentration of AS, however, was not identical to the concentration of ions in HS, so HS/AS was prepared to have (almost) the same protein content as HS, but with the same mineral ion concentrations as AS. This would allow a direct comparison of the effect of mineral ions alone (AS) to that of mineral ions together with proteins (HS/AS).

We observed high initial SH values (SH_0_), which decreased in all groups as the experiment progressed. As expected, the greatest decreases in SH were observed immediately after erosive challenges (SH_2_, SH_4_, SH_6_, and SH_8_). After the saliva incubation periods following erosions, slight increases in SH were observed in all groups, except for the Ctrl group. The greatest increases occurred in the HS group, but the SH values obtained before the preceding erosion steps were never reached again. Moreover, this effect was not enduring, as the SH values of the HS group after erosion decreased to a similar level as those of the AS group, and at the end of the experiment we found no significant difference in SH values between the HS and AS groups.

A possible explanation for the comparably high increase in SH values after incubation in the HS group is that ions formed mineral deposits on the eroded enamel surface. The reason for this deposition could be twofold: it could be due to the presence of the ions themselves in the solutions, and it could be due to the presence of some proteins in the solutions. HS contains a comparatively high phosphate concentration, as well as other ions that were removed in the other groups by dialysis. These different ion concentrations and compositions (Table 1) might explain the differences observed in the results. A comparison of HS/AS and AS groups supports this thesis. They have the same ion composition, and we observed no great differences in the increase in SH values after the incubation periods in those groups. In addition to calcium and phosphate, other ions also affect the dissolution of hydroxyapatite (HAp)^14^. It has been suggested that chloride and sodium are also associated with suppression of HAp dissolution, possibly as a result of competition for HAp surface protonation sites between Na^+^ and H^+^ ions, but not by the incorporation of Cl^−^ ions into HAp^14^. Therefore, the presence of all these ions is probably an important factor in the increase in SH of enamel.

Another factor that is involved in increasing the SH values is the presence of some proteins. Remarkably, the solution deprived of free ions (HS/DW group) also showed a slight increase in hardness after incubations after erosions, which must mainly be due to the proteins present in the solution. Different proteins have been shown to either inhibit or promote erosion or lesion remineralization^27^,^28^,^29^. Also, faster adsorption of proteins to eroded enamel has been reported^30^. The influence of proteins on enamel demineralization varies significantly, depending on how much is adsorbed and how much free calcium is available near the surface^31^. The balance of enamel porosity, the degree of saturation of a solution with respect to enamel minerals, and protein concentrations in the solution seem to determine the exact effect that the solution has on the enamel surface^28^. This could explain the SH pattern we observed in the case of the HS/DW group, which seemed to have a slight rehardening effect on eroded enamel, but the opposite effect on polished enamel with a slight decrease in hardness after pellicle formation (SH_0_–SH_1_, Fig. 1). The exact mechanism underlying how SH can increase after an incubation with a solution containing primarily proteins but no meaningful amounts of ions is not clear and warrants further investigation. One possible explanation might be that salivary proteins penetrate into enamel pores opened by erosion, acting as bridging ligaments between adjacent enamel rods and thereby toughening the enamel, which has been reported for enamel proteins^32^.

In addition to their effect on SH modulation, the proteins are most importantly responsible for the formation of the salivary pellicle, which also plays an important role in the protection against erosion. This was mainly observed in the HS/DW group, where there were very little ions present, and the incubation solution was mostly made up of proteins. The salivary pellicle formed with HS/DW presented the best protective effect in our study, with the least overall decrease in SH and the least calcium release (Figs 2 and 4). Our results, however, are not consistent with the results obtained in the study by Martins *et al*.^33^, which showed that dialysed saliva did not provide good protection against enamel demineralisation and that whole saliva provided the most effective erosion protection. These authors carried out only 16 h of dialysis, whereas we dialysed for 72 h, so our method might have removed the mineral ions more thoroughly. Furthermore, the authors measured the effect only with respect to calcium and phosphate release, not with respect to hardness. Owing to reasons explained below, our own calcium release measurements did not allow for a comparison of the undialysed HS with HS/DW, so a direct comparison of our results to the results of Martins *et al*. is not possible.

Recently, it was suggested that the organic components (proteins) of saliva, either solely or by interacting with salivary chemical properties, could have a significant impact on the susceptibility of the tooth surface to demineralisation^8^. In case of HS/DW, most of the ions, especially calcium and phosphates, were removed from the saliva. When calcium and phosphates are present in saliva, they will compete for the binding sites on calcium- and phosphate-binding proteins with the ions present on the enamel surface. Lacking calcium and phosphate ions, we speculate that the calcium- and phosphate-binding proteins in HS/DW could bind to the enamel surface better. Hence, the salivary pellicle basal layer formed by HS/DW was probably more strongly bound to the enamel surface, and therefore more protective against erosion. In other words, while being indispensable to allow remineralisation of dental hard tissues, calcium significantly affects the protective effect of the salivary pellicle, and its concentration has been shown to have a dramatic impact on pellicle thickness and density^34^. HS and HS/AS presented greater protein content, as well as greater calcium and phosphate concentrations, but they exhibited a significantly less protective effect than HS/DW.

With regard to the calcium release results, we observed that the HS, AS and HS/AS groups released similar amounts of calcium throughout the experiment (Figs 3 and 4). Usually, this technique is used to measure the amount of calcium from dental hard tissues that dissolves into the citric acid during an erosive challenge^35^. The Ctrl group, being unprotected, was expected to exhibit the greatest calcium release. However, the HS, AS and HS/AS groups released larger amounts of calcium than the Ctrl group. Since the entire specimens were immersed in the incubation solutions, a pellicle and/or calcium deposits also likely formed on the resin surfaces and not only on the enamel surfaces. The calcium release values measured therefore originate not only from the dental hard tissue, but also from the pellicle itself or from calcium deposits formed during the incubation time. In this respect, other studies have shown a considerable amount of calcium released from the saliva film formed on porcelain discs^14^. Due to limitations in the amount of saliva available, we were not able to determine the amount of calcium released from deposits or pellicles formed on resin surfaces. Hence, we could not correct the calcium release values for this factor. Given an overall surface area of ~660 mm^2^ for the specimens, with an average exposed enamel area of only 12.8 mm^2^, the amount of calcium released from deposits or pellicles formed on the resin surface can surely not be neglected. Therefore, the calcium release results for the three groups HS, AS and HS/AS did not allow us to draw any conclusions.

However, we can still draw conclusions from the Ctrl and HS/DW groups. The Ctrl group calcium release values can originate only from the apatite crystals of the enamel. Considering that HS/DW contained only 0.15 mmol/l of calcium, the amount of calcium bound by the pellicle in this group was negligible, so the calcium released mainly derived from the tooth surface. However, the HS/DW group clearly showed the least amount of calcium loss, and this was observed from the first cycle (1 min) onward (Fig. 3). It released significantly less calcium than the Ctrl group (Fig. 4), which means that the pellicle formed in this group was able to significantly protect the enamel from erosion. Interestingly, the lower amount of calcium released is also consistent with the SH results, where samples incubated in HS/DW presented the least SH decrease (least erosion).

Although *in vitro* results are difficult to apply to *in vivo* circumstances, it would be interesting to know to what extent we can extrapolate these study results to the clinical situation. We observed an increase in SH during the 1 h incubation in human saliva in our experiment. However, *in vivo* remineralisation is quite a slow process^8^. Additionally, contrary to subsurface caries lesions, any remineralisation of erosion lesions is restricted to the softened near-surface demineralised enamel layer^5^,^19^. Even if some enamel remineralisation occurred in our samples, it did not completely protect the enamel against further demineralisation. Furthermore, any remineralisation from saliva occurring *in vivo* during the short time between acid attack and toothbrushing would also not protect the enamel against substance loss from abrasive forces^19^. Therefore, any increase in SH observed in our study would not lead to clinically relevant protection, and the focus should be more on protection from erosion rather than on the seemingly unsustainable remineralization effects of saliva after erosion.

## Conclusion

Salivary proteins as well as salivary ions both play a role in protecting enamel from erosion. Pellicles formed by salivary proteins depleted of salivary ions (HS/DW) provide the best erosion protection effect, as indicated by the least amount of decrease in SH and the least amount of calcium released throughout the entire experiment. This might be due to a stronger binding of calcium- and phosphate-binding proteins to the enamel surface in the absence of free ions. Incubation in AS, containing only salivary ions, and pellicles formed by HS provided slightly less protection, and no significant differences were observed between the respective treatment groups. If salivary proteins and ions are both present, the exact composition appears to be important for the erosion protection effect, as demonstrated by the differences between the HS and HS/AS groups.

## Additional Information

**How to cite this article**: Baumann, T. *et al*. Erosion protection conferred by whole human saliva, dialysed saliva, and artificial saliva. *Sci. Rep.* **6**, 34760; doi: 10.1038/srep34760 (2016).

## Figures and Tables

**Table 1:** Calcium, phosphate and protein concentrations of the incubation solutions.

Group	Incubation solution	Dialysis method	Calcium (mmol/l)	Phosphate (mmol/l)	Protein (μg/ml)
Ctrl	Humid chamber	—	—	—	—
HS	Human saliva	—	1.23	3.53	895.85
AS	Artificial saliva*	—	1.56	0.85	—
HS/AS	Human saliva	Artificial saliva*	1.52	0.75	738.24
HS/DW	Human saliva	Deionised water	0.15	0.10	675.13

*1.5 mM Ca(NO_3_)_2_, 0.90 mM KH_2_PO_4_, 130 mM KCl and 60 mM Tris buffer; pH 7.4 21.
